# Coupling of receptor interference and a host-dependent post-binding entry deficiency in a gammaretroviral envelope protein

**DOI:** 10.1186/1742-4690-7-9

**Published:** 2010-02-05

**Authors:** Shervin Bahrami, Ditte Ejegod, Karina Dalsgaard Sørensen, Finn Skou Pedersen

**Affiliations:** 1 Department of Molecular Biology, Aarhus University, DK-8000 Aarhus, Denmark; 2 Interdisciplinary Nanoscience Center (iNANO), Aarhus University, DK-8000 Aarhus, Denmark

## Abstract

**Background:**

SL3-2 is a unique polytropic murine gammaretroviral isolate that is only able to infect murine cells. We have previously shown that two mutations R212G and T213I located on the surface of the receptor binding domain in a region designated the VR3 loop can alter the species tropism of this envelope protein. This location suggests that the VR3 loop composition has an influence on receptor interaction and thereby affects binding as well as superinfection resistance. In order to investigate this further, we have studied the binding and interference patterns of the SL3-2 envelope and its mutants.

**Results:**

We find unexpectedly that wild type SL3-2 envelope binds equally well to both permissive and non-permissive cells, indicating a post binding defect when interacting with the human Xpr1. Using replication competent viruses containing envelopes from SL3-2 or its mutants we find that the same amino acid mutations can dramatically alter the interference profile of this polytropic ENV, suggesting that the same amino acid changes that cause the post binding defect also influence interaction with the receptor.

**Conclusions:**

The envelope protein of SL3-2 MLV shows an entry defect on non-murine cells. This is coupled to a dramatically reduced ability to interfere with entry of other polytropic viruses. Two point mutations in the VR3 loop of the receptor binding domain of this envelope result both in a much increased interference ability and in removing the post-binding defect on non-murine cells, suggesting that both of these phenotypes are a consequence of insufficient interaction between the envelope and the receptor

## Background

Retroviruses enter their host cells by binding to specific cellular proteins followed by fusion of viral and cellular membranes. This interaction is mediated by the viral envelope glycoproteins. The envelope protein is a single gene product that is post-translationally cleaved to give a surface subunit (SU), which is responsible for binding to the receptor, and a transmembrane subunit (TM) that mediates the fusion of viral and cellular membranes.

The surface subunits of different viruses have evolved to use different cellular proteins as receptors. Even among closely related viruses, receptor usage can be markedly different. This is indeed the case for gammaretroviruses of murine and other species. At least five different receptors have been identified for this group including mCAT1 [1-3], Pit1 [4], Pit2 [5], Xpr1 [6-8] and Smit1 [9]. The greatest variation in species tropism is found in viruses that use Xpr1 as receptor. Within this group, xenotropic viruses can infect cells of different species but not those of murine origin, while polytropic viruses can use the Xpr1 receptor on cells of many different species including murine cells. Finally SL3-2 is a unique isolate of the polytropic virus group able to infect only murine cells [10,11]. The determinant for the limited species tropism of SL3-2 was mapped to two amino acids in an exposed loop, the VR3 loop, in the receptor binding domain of the surface subunit not previously implicated in receptor interaction. Thus, mutation of arginine 212 and threonine 213 to glycine 212 and isoleucine 213, enables the SL3-2 envelope to efficiently use the human and mink Xpr1 without compromising its infectivity towards murine cells [10].

To address the step in the entry process where this determinant of species tropism exerts its function we have investigated the binding of SL3-2 to non-permissive cells and the interference pattern of wild type SL3-2 virus and its VR3 mutants on permissive murine cells. Interference is the phenomenon of blocking entry receptors of a cell by envelope proteins produced within the same cell. In other words, an envelope protein expressed in a cell can bind to its receptor and thereby prevent infection by viruses that use the same protein as receptor [12]. Among the viruses that use the Xpr1 receptor, those of the polytropic virus group have been found to exhibit incomplete interference [13,14].

We find that wild type SL3-2 can bind to non-permissive cells and thus has a post binding defect on human cells. At the same time, this envelope is much less efficient in interfering with viruses that use Xpr1; whereas the R212G, T213I mutant (that has a wider tropism) shows nearly complete interference. Since interference is a consequence of receptor binding independent of the fusiogenic activity of the envelope [15], we interpret these data as evidence for a direct or indirect contribution of the VR3 loop to interaction with the Xpr1 receptor. Interestingly, polytropic and xenotropic viruses show similar non- reciprocal interference, suggesting that these viruses bind with different affinities to different parts of the Xpr1 receptor [13]. To our knowledge, this is the first example of an interaction between gamma-retroviral envelope and its receptor(s), which primarily affects activation of the fusion machinery in a receptor dependent manner.

## Results

### SL3-2 envelope binds to non-permissive human cells

**Figure 1:**
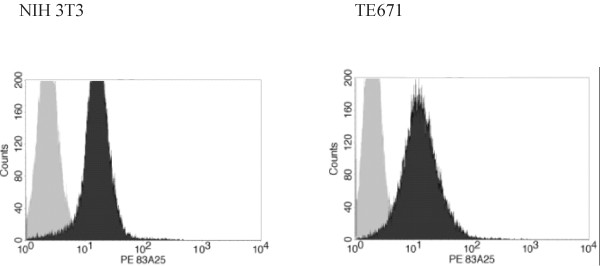
**Binding of virions containing the envelope protein of the SL3-2 MLV to permissive (NIH 3T3) and non-permissive (TE671) cells**. The cells were incubated with supernatants from cultures that either did or did not produce viral particles at 4°C and subsequently labeled with anti-envelope antibodies for visualization in a flow-cytometer. (data are representative of several experiments). Light grey: supernatant containing no virions, dark grey: virion containing supernatant.

As evident from Fig. 1, virions containing the wild type mouse tropic SL3-2 envelope protein are still able to bind to the non-permissive human cells in a receptor dependent binding assay [16,17]. Hence the SL3-2 envelope has a post-binding fusion deficiency on non-murine cells. As such it is similar to another fusion deficient variant of MLV envelope in which the H8R mutation renders the protein non-fusogenic, but still able to bind to permissive cells [17,18]. The residue at position 8 plays an important role in fusion activation regardless of the receptor. A retargeted envelope protein shows the same dependency as wt envelopes on this critical residue [19]. The major difference between the phenotype caused by mutations at positions 212 and 213 in the SL3-2 envelope protein and histidine 8 mutants is that in the case of SL3-2, the fusion deficiency is dependent on the target cell and therefore most likely on the viral receptor.

In this case, the VR3 loop should affect the interaction between the envelope and receptor either by direct binding or by causing conformational changes in the overall envelope structure so as to affect this interaction non-reciprocally. To clarify this issue, we wished to analyze the interference properties of the wild type or the VR3 mutants of the SL3-2 envelope protein. Since interference is solely dependent on receptor binding, any change in the mutants' ability to cause interference is direct evidence for alterations in binding to the receptor caused by mutations in the VR3 loop.

### Replication competent SL3-2 viruses

**Figure 2:**
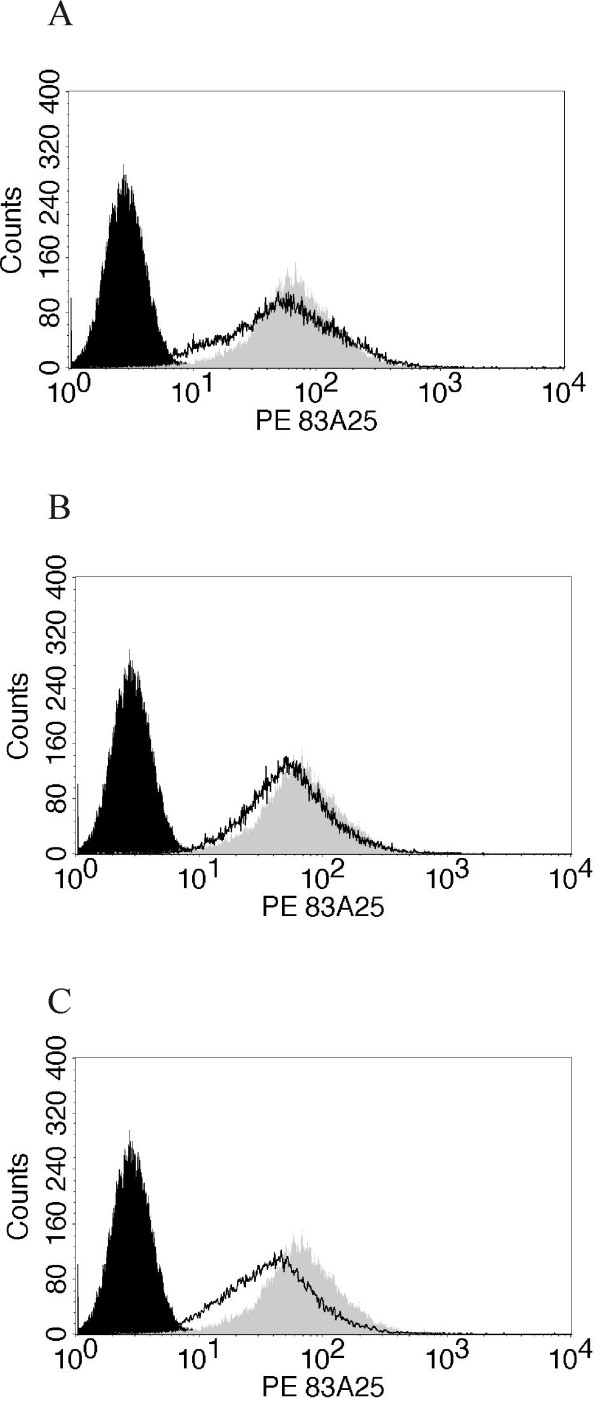
**Flow cytometric analysis of viral expression using anti-envelope antibodies**. Filled black: NIH3T3 (negative control), filled grey SL3-3 (positive control). A) SL3-2 wildtype, B) SL3-2 GI and C) SL3-2 MV.

Measurement of relative interference properties requires a sufficient level of envelope protein expression in all target cells to allow for determinations of relative infectivities above a background of infection of cells with low or no envelope expression. This may be particularly important in the case of polytropic viruses which have been reported to exhibit incomplete interference properties [13,14]. We therefore constructed replication-competent viruses that contain wild type or VR3 mutants of SL3-2 envelope protein for use in interference experiments. SL3-2 virus was isolated from a tumor cell line from AKR mice, but no molecular clone of the original virus exists, only the envelope gene has been cloned [10,20]. Since the genome of SL3-2 is very similar to SL3-3 virus except for the envelope gene [20], a replication competent virus containing the former envelope was constructed by replacing the envelope gene of a cloned SL3-3 virus with that of SL3-2 or its VR3 mutants. As can be seen in Fig. 2, NIH 3T3 cells infected with these viruses constituted a uniformly infected population with identical envelope expression levels. To confirm the presence of the intended virus, RNA was isolated from supernatants used for RT-PCR using envelope primers, and the resulting fragments were sequenced (data not shown). These cells were used for determination of interference in the subsequent studies.

### The role of the VR3 loop in receptor blocking

In order to facilitate titer measurements, we used bi-cistronic vectors that contain both a neomycin resistance marker and the envelope gene. Vectors containing envelopes from wt SL3-2, SL3-2 GI, SL3-2 MV or MCF 247 envelope genes were constructed previously as described [10]. Glycine and isoleucine in the VR3 loop are found in polytropic viruses (including MCF 247) that have a wide tropism. Methionine and valine in VR3 loop have been selected for infectivity of human cells from a randomized library. Both mutants infect human and mink cells with wild type efficiency [10].

The vectors were co-transfected into 293T cells together with a gag-pol expression plasmid. The supernatants from these cells were used to transduce semi-packaging cells which are derived from NIH 3T3 and stably express Gag-Pol polyprotein from Moloney murine leukemia virus. The semi-packaging cells were selected with G418 until a resistant population emerged. These cells produce virions with the respective envelopes containing the bi-cistronic vector. The supernatants from semi-packaging cells were used for titer measurements on the NIH 3T3 cells stably transduced by replication competent SL3-2 viruses or NIH 3T3 cells containing MCF 247 virus. As negative control, we used NIH 3T3 cells infected by the SL3-3 virus which uses the ecotropic mCAT1 receptor [21] and does not interfere with entry via Xpr1.

**Figure 3:**
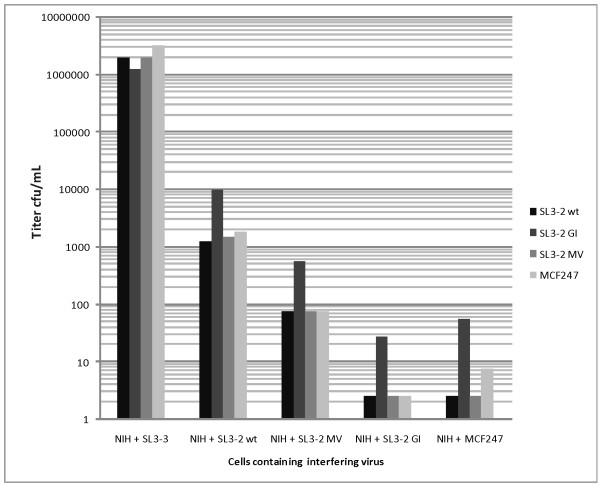
**Interference pattern of different VR3 mutants of the SL3-2 Envelope protein**. Murine cells chronically infected with replication competent viruses were infected with virions containing different envelope mutants as well as a neo selection marker. 24 h post infection the cells were selected by G418 until emergence of colonies. The figure shows the results for one of two independent experiments yielding smiliar results.

As can be seen in Fig. 3, the wt SL3-2 envelope is less efficient in receptor blockage than are both SL3-2 MV and SL3-2 GI, suggesting that the latter two envelopes bind more strongly to the receptor than wt SL3-2. Interestingly, MCF247 also contains glycine and isoleucine in the VR3 loop and shows as efficient interference as does the SL3-2 GI construct. Virions containing SL3-2 GI envelope protein seem to be less sensitive to receptor blockage by other polytropic viruses (with titers around one order of magnitude larger than the other viruses). This is consistent with the view that this envelope protein interacts more strongly with the receptor than wt SL3-2. Although these data do not exclude the possibility of interaction with a co-receptor, no direct evidence for existence of one for murine gammaretroviruses exists. Thus, glycine and isoleucine in the VR3 loop of the envelope protein confer both an efficient superinfection resistance and also seem to enable the virus to bypass receptor blockage slightly more efficiently. Together this suggests that SL3-2 GI envelope has a stronger interaction with the murine Xpr1. The methionine and valine mutant SL3-2 MV that has originally been selected from a library for infectivity on human cells [10] is inferior to SL3-2 GI in blocking the receptor. These amino acid residues are not found in VR3 loops of any known wild type viruses. The efficiency of MCF247 (which has glycine and isoleucine in its VR3 loop) in conferring interference is expectedly comparable to SL3-2 GI, but at the same time it seems that SL3-2 GI is more capable of bypassing receptor blockage suggesting better efficiency of infection than that of MCF247. This is in agreement with our previous observations that SL3-2 GI has a higher titer on both mink and human cells than MCF247. Although there is 93% identity between SL3-2 and MCF247 envelope sequences, there is only 80% identity between the VRB regions of these two envelopes, suggesting involvement of this region in receptor interaction. An alignment of the envelope sequences can be found in [10].

## Discussion

Murine gammaretroviruses that use Xpr1 as receptor form a relatively diverse group, with significant differences in species tropism and non-reciprocal interference patterns. Specifically, xenotropic and polytropic viruses that both use the Xpr1 receptor seem to have affinity for at least two different extracellular loops of the same receptor [13,14,22]. The exact binding surface on the polytropic/xenotropic/SL3-2 envelopes is not known, but the N-terminal segment of SU containing the variable region A (VRA) plays a role [23]. Binding to more than one loop on the receptor suggests that these viral envelopes must contain an accordingly large binding site on their SUs; therefore, other sites on the envelope may play roles. Here we present data that enforce our previous observations [10] that the composition of the VR3 loop influences the interaction with Xpr1.

The VR3 loop has previously been shown to be involved in infectivity of human cells [10], but it was not known whether this was because of receptor binding or post-binding steps. The facts that wt SL3-2 envelope (with arginine and threonine in VR3 loop) can bind to non-permissive cells together with inferring a much reduced ability to block Xpr1 (on permissive murine cells) from interaction with other viruses, suggest that the VR3 loop affects the interaction between the envelope and its receptor. One possibility is a direct binding of VR3 to Xpr1. In this case, either this interaction is not essential for successful infection of murine cells or VR3 loops containing arginine and threonine can only bind to the murine Xpr1. On human cells on the other hand, it is conceivable that binding of wild type SL3-2 is not strong enough without a positive contribution of the VR3 loop to result in viral entry. Another possibility is that the VR3 loop of the wt SL3-2, through steric hindrance, causes inefficient binding to non-murine Xpr1 receptors, which in not enough to activate the fusion machinery of the envelope protein to allow entry. Alternatively, the presence of different amino acid residues in VR3, can cause conformational changes in the rest of the envelope in a way that affects its binding efficiency to Xpr1, causing the aforementioned phenotypes.

It would be interesting to investigate whether SL3-2 ENV, even though it is not able to use non-murine XPR1 receptors, can cause interference with other polytropic viruses in non-permissive cells. To test this, we cloned wt SL3-2 and SL3-2 GI mutant into an expression vector previously used for expression of high levels of MLV envelope protein [24]. The resulting plasmids were transfected into TE671 cells and selected with puromycin until a resistance culture emerged that expresses these envelope proteins. However, the expression levels of ENV from these constructs were not high enough to show any superinfection resistance. The notion that repeated infections by a replication competent virus are necessary for efficient interference to emerge in the case of polytropic viruses is in agreement with the literature [11,13,14]. This is in contrast to interference caused by vector expression of the ecotropic envelope protein in mouse cells where at least four orders of magnitude of titer reduction of an incoming ecotropic vector particle can be observed [10].

Interference occurs naturally in some mouse strains as a defense against polytropic viruses (e.g. a xenotropic provirus confers resistance to polytropic viruses in *Mus castaneus *[25]). Interestingly, the endogenous viral envelope that causes resistance to polytropic murine leukemia virus infections in DBA/2 mice contains glycine and threonine in the VR3 loop. Interference from this envelope is only two orders of magnitude (which is comparable to interference from SL3-2); yet it is enough to inhibit spreading of polytropic viruses in these mice [26].

**Figure 4:**
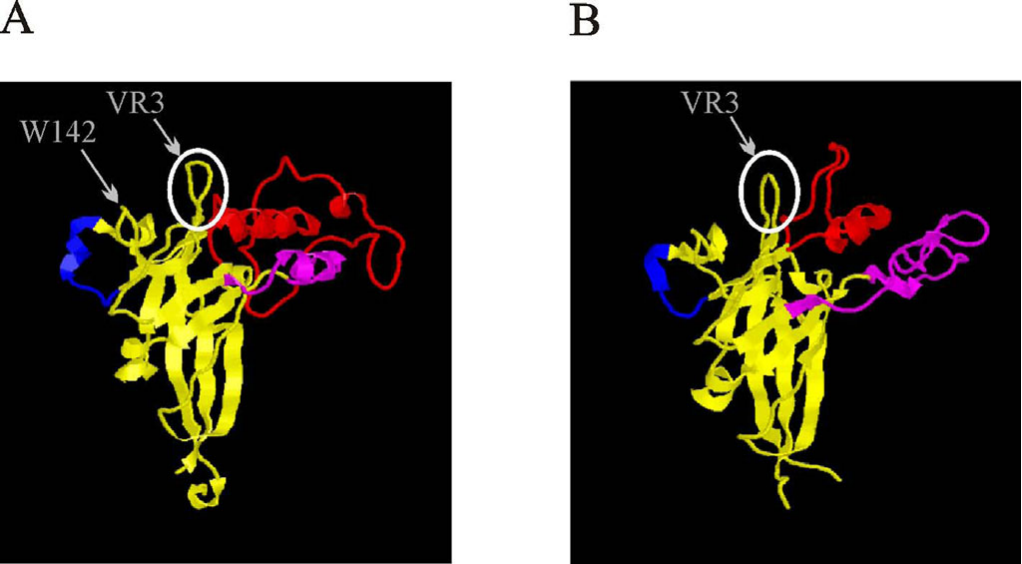
**Crystal structure of the receptor binding domain of the Friend murine leukemia virus (A) and FeLV-B (B)**. VRA, B and C loops are shown in red, purple and blue. VR3 loop is encircled. The pictures are generated using the Rasmol software and coordinates (A: 1AOL B: 1LCS) from the RCSB protein data bank http://www.rcsb.org/pdb/home/home.do.

Evidence from ecotropic and amphotropic viruses suggest that VRA and VRB regions are directly involved in interaction with the receptors. These two variable regions as well as VR3 constitute three loops located in close proximity on the surface of the SU subunit as shown in crystal structures of receptor binding domains of related envelope proteins of Friend murine leukemia virus [27] and FeLV-B [28] (Fig. 4). It is therefore possible that VRA, VRB and VR3 loops constitute a binding surface that interacts with the extracellular loops 3 and 4 of the Xpr1. According to such a model, a certain affinity between the binding surfaces of the envelope and the receptor is necessary for triggering the fusion potential of TM, but how this affinity is distributed between the three different loops is less important. The non-reciprocal interference patterns observed in polytropic and xenotropic viruses [13] and the different roles that VR3 loop plays in binding to human or mouse Xpr1, would then arise from the varying degrees with which VRA, VRB and VR3 loops contribute to the overall binding affinity between envelope and receptor.

The receptor binding site of the ecotropic envelopes seems to involve residues located in VRA [29]. It is noteworthy that VRA in ecotropic envelopes is considerably larger than in other murine leukemia virus isolates. Interestingly, a conserved tryptophan (W142, see Fig. 4) on the same surface of the protein as the VR3 loop has also been implicated in receptor interaction [30], supporting the idea that a large surface of the envelope protein must interact with the receptor to facilitate viral entry. VR3 loop is flanked by W142 and VRA loop, and the potential role for it in ecotropic viruses remains to be investigated.

Overall our data suggest that the interaction between the gammaretroviral envelope protein and its receptor, at least in the case of the polytropic murine leukemia viruses, is affected by the small VR3 loop. This interaction is not critical for viral binding and is probably less important than the interaction between the VRA and Xpr1 for viral entry. However, amino acid substitutions in the VR3 loop may modulate species tropism as well as receptor interference pattern. This is an example of an interaction between an envelope protein and its receptor(s), which primarily affects activation of the fusion machinery in a receptor dependent manner. The results thereby suggest that simple binding to a receptor protein is not sufficient for a successful fusion event, but the interaction between the envelope protein and its receptor must fulfill as yet undefined structural requirements for activation of the conformational changes that result in membrane fusion.

## Methods

### Replication competent viruses

It has previously been shown that SL3-2 and the ecotropic SL3-3 are closely related outside env [20]. Since no complete clone of SL3-2 exists, replication competent viruses were constructed by cloning of SL3-2 envelope proteins into the SL3-3 MLV using standard cloning techniques. The resulting viral genomes were transfected into 293T cells and 48 h post transfection, supernatants were transferred to NIH 3T3 cells. Successful infection was confirmed by FACS. The MCF247 expressing NIH cells were acquired separately [31].

### RT-PCR

Supernatant from virus-producing cells was ultra-centrifuged for 90 minutes at 4°C and 25,000 rpm to pellet the viral particles using an Optima L 80XP centrifuge and SW41 rotor. The pellet was resuspended in 500 μl TRIzol^® ^reagent (Invitrogen) and RNA purified as described by the manufacturer. cDNA was subsequently produced using First-Strand cDNA Synthesis Kit (GE Healthcare). 1-5 μg RNA and specific SL3-2 *env *reverse primer (215066: 5'CGGGTCGGGAGGGGGGTAACT 3') was used to produce cDNA, which was subsequently amplified by PCR using SL3-2 *env *specific primers (215066 and 212504: 5'TGCTAGCAGGGTGTGGAGGGC 3'). PCR fragments were purified and sequenced using BigDye^® ^Terminator v3.1 Cycle Sequencing Kit, using primer 215066 and 212504 (Applied Biosystems).

### Flow cytometry analysis

Cells were released from plates, washed twice using PBS with 2% serum and incubated with 500 μl of supernatant from the 83A25 [32] hybridoma cell line in 4°C for 45 minutes. The cells were subsequently washed twice, and 5 μl of 1:40 diluted secondary antibody (Goat anti-Rat Ig R-PE conjugate, Harlan Sera-Lab) were added to the cell pellet. After incubation at 4°C for 45 minutes, they were washed twice in PBS with 2% serum and suspended in a 1% formalin solution and analyzed by a Becton Dickinson FACSCalibur cytometer.

### Virus binding assay

Virions were concentrated on a Centricon Plus-80 (100-kDa cutoff) column and incubated with target cells at 4°C for 45 minutes. The cells were washed twice using PBS with 2% serum and incubated with 500 μl of supernatant from 83A25 [32] hybridoma cell line in 4°C for 45 minutes. The cells were subsequently washed twice, and 5 μl of secondary antibody (Goat anti-Rat Ig R-PE conjugate, Harlan Sera-Lab) were added to the pellet of cells. After incubation at 4°C for 45 minutes, they were washed twice in PBS with 2% serum and suspended in a 1% formalin solution and analyzed by a Becton Dickinson FACSCalibur cytometer.

### Titer measurements

Viral particles produced from semi-packaging cell lines transduced by bi-cistronic vectors [33] bearing both *neo *and *env *genes of SL3-2 wt, SL3-2 GI mutant, SL3-2 MV mutant, or MCF 247 [10] were transferred to target cells in serial 10-fold dilutions. After 25 h, the cells were subjected to selection in medium containing 600 μg/mL of G418 until colonies appeared. Titer data were confirmed in two independent experiments.

## Competing interests

The authors declare that they have no competing interests.

## Authors' contributions

SB helped design the study, carried out the binding and interference experiments, analyzed the data and composed the manuscript. DE and KDS devised the cloning strategy of the replication competent viruses and performed the cloning. FSP helped design the study and write the manuscript.

## Acknowledgements

We would like to thank Ane Kjeldsen and Lene Svinth Jøhnke for technical assistance and Alexander Schmitz for help with flow cytometry. This work is supported by The Danish Agency for Science, Technology and Innovation, The Lundbeck Foundation, Fonden til Lægevidenskabens Fremme, and The Danish Cancer Society. Ditte Ejegod is a fellow of the Research School in Gene Medicine.
